# Waste Material Classification: A Short-Wave Infrared Discrete-Light-Source Approach Based on Light-Emitting Diodes

**DOI:** 10.3390/s24030809

**Published:** 2024-01-26

**Authors:** Anju Manakkakudy, Andrea De Iacovo, Emanuele Maiorana, Federica Mitri, Lorenzo Colace

**Affiliations:** Department of Industrial, Electronic and Mechanical Engineering, Roma Tre University, 00146 Rome, Italy; anju.manakkakudykumaran@uniroma3.it (A.M.); emanuele.maiorana@uniroma3.it (E.M.); federica.mitri@uniroma3.it (F.M.); lorenzo.colace@uniroma3.it (L.C.)

**Keywords:** discrete spectroscopy, feature selection, SWIR, material classification, optical sensor

## Abstract

Waste material classification is a challenging yet important task in waste management. The realization of low-cost waste classification systems and methods is critical to meet the ever-increasing demand for efficient waste management and recycling. In this paper, we demonstrate a simple, compact and low-cost classification system based on optical reflectance measurements in the short-wave infrared for the segregation of waste materials such as plastics, paper, glass, and aluminium. The system comprises a small set of LEDs and one single broadband photodetector. All devices are controlled through low-cost and low-power electronics, and data are gathered and managed via a computer interface. The proposed system reaches accuracy levels as high as 94.3% when considering seven distinct materials and 97.0% when excluding the most difficult to classify, thus representing a valuable proof-of-concept for future system developments.

## 1. Introduction

Material classification plays a crucial role in various industries such as manufacturing, healthcare, environmental monitoring, and security. The ability to accurately identify and classify different materials is essential for ensuring product quality, safety, and compliance with industry standards. In recent years, the importance of material classification has been further amplified by the growing emphasis on sustainability and environmental conservation [1].

In this era of rapid technological advancements, sensors have emerged as indispensable tools for material classification. Unlike traditional methods that often rely on manual inspection or chemical analysis, sensors offer real-time, non-destructive, and precise material identification capabilities. For this aim, various technologies such as spectroscopy, imaging, and X-ray analysis can be employed [2]. Among such approaches, those relying on optical sensors stand out, due to their ability to use infrared light to gather information about material characteristics that are often invisible to the human eye. Optical sensors offer remarkable precision and speed in distinguishing materials, without the need for extensive sample preparations or prolonged laboratory tests [3]. Moreover, these sensors operate in non-contact mode, facilitating the analysis of moving materials.

The integration of optical sensors with cutting-edge technologies such as hyperspectral imaging and Raman spectroscopy has further elevated their capabilities. Machine learning algorithms, fuelled by data collected from optical sensors, enable intelligent pattern recognition, resulting in highly accurate material classification outcomes [4]. Industries spanning pharmaceuticals, food processing, aerospace, and environmental monitoring have significantly benefited from the advanced capabilities of optical sensors [5,6,7].

Optical sensors typically exploit spectral information to identify materials based on their reflectance and/or transmittance fingerprint. Different spectral ranges can be employed, depending on the materials of interest and on the morphological characteristics of the samples. Short-wave infrared (SWIR) spectroscopy stands out as a beacon of precision. Operating within the wavelengths from 1000 to 2500 nm, SWIR spectroscopy offers an unparalleled depth of analysis. Its ability to penetrate materials, coupled with its sensitivity to various molecular vibrations, makes it indispensable in material classification [8]. SWIR spectroscopy and SWIR-based hyperspectral imaging have been extensively employed in the last decade for material classification purposes, when used together with classification algorithms [9,10,11].

Spectral analysis and hyperspectral imaging can reach very high performance in material classification, but, on the other hand, they rely on cumbersome and expensive instrumentation, thus hampering their adoption for high-volume applications. Recently, a simplified approach to spectral measurements has been proposed, employing imaging systems and photodetectors with a large spectral response and quasi-monochromatic light sources (LEDs), selected in order to investigate only those wavelengths where target materials express specific reflectance signatures [12,13,14]. The extension of such a discrete-light-source spectroscopy approach to SWIR wavelengths can further enhance the achievable performance, allowing for better material recognition.

In this study, we explore SWIR-based material classification using discrete LED sources for spectral analysis. Our preliminary study utilized 10 carefully chosen LEDs, achieving 98% accuracy in classifying waste materials [15]. The current research aims to further improve the proposed system by revisiting the initial set of LEDs and optimizing the achievable accuracy while reducing the number of employed LEDs. The proposed system measures the reflectance of solid samples at specific wavelengths, acquiring discrete reflectivity spectra while minimizing data redundancy. The goal is to create a cost-effective, user-friendly instrument for material sorting, applicable in recycling and waste management. Despite potential performance trade-offs, our approach ensures quick and efficient implementation, making it ideal for distributed waste segregation, enabling the accurate differentiation of waste materials.

**Figure 1:**
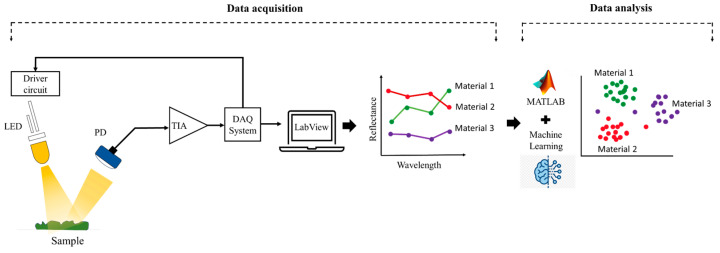
Schematic representation of the system working principle.

Figure 1 presents a schematic overview of the principle of operation of the system. In this configuration, LEDs function as light sources, sequentially controlled by electronic drivers, while the photodetector measures the reflected intensities. These signals undergo conditioning through a trans-impedance amplifier, before being acquired through a commercial data acquisition interface. The MATLAB suite is employed for the classification of the acquired data, corresponding to different materials.

## 2. Materials and Methods

### 2.1. Materials

In our study, we analysed seven materials, including polyethylene terephthalate (PET), polypropylene (PP, transparent and white), a composite polymer made of polylactic acid (PLA) and polybutylene succinate (i.e., AB400L), glass, paper, and aluminium. It is important to note that these samples represent only a subset of the materials found in industrial and domestic waste, and they were utilized in the forms of flakes and pellets, which might not fully represent real waste materials in terms of physical properties. Hence, our system serves as a proof of concept, demonstrating the potential of discrete spectroscopy for waste segregation. We specifically selected transparent or white materials as reference samples, except for light-grey PET, since SWIR spectroscopy still poses challenges in analysing dark-coloured samples due to their high absorption levels [16]. In addition, cutting the materials into macro-sized flakes provided several advantages, such as an increased sample surface area, material homogenization, and reduced effects of extraneous reflections like specular reflections.

### 2.2. SWIR Spectral Acquisition

**Figure 2:**
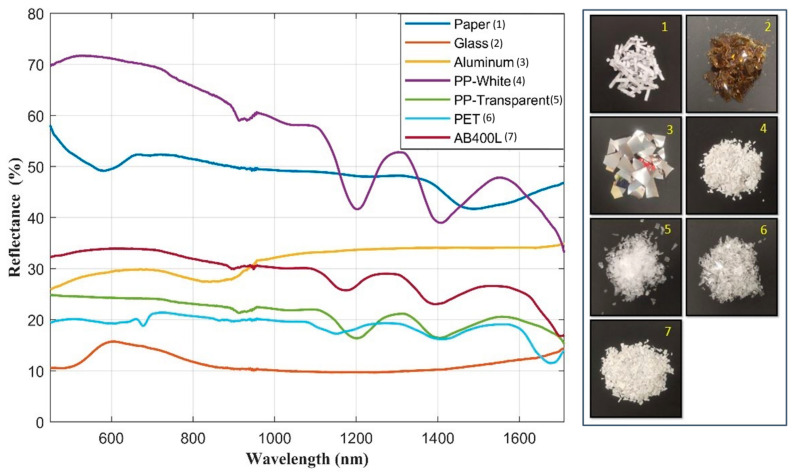
Spectra of all materials using compact spectrometers. The numbers in the photographs align with the labels provided in the plot legends.

The spectra for each material were collected using two distinct Ocean Insight compact spectrometers (Orlando, FL, USA) operating in the VIS (450 nm to 1030 nm) and SWIR (954 nm to 1710 nm) ranges, with a step size of 1 nm. Subsequently, the individual VIS and NIR spectra were rescaled, merged, and presented as a unified spectrum spanning the range from 450 nm to 1710 nm. A total of 50 spectra were recorded for each material. Figure 2 illustrates the combined averaged reflectance spectra obtained from the two compact spectrometers. The right side of the figure shows photographs of each material.

The reflectance spectra reveal that glass distinguishes itself from other materials by virtue of its exceptional transparency, characterized by a solitary peak in the 600 nm wavelength range. The white PP material exhibits elevated reflectance values in comparison to other materials, featuring two prominent peaks within the wavelength range of 1200 nm to 1600 nm. Transparent PP shares peaks with white PP, but its reflectance values are significantly lower, and the peaks appear broader. The paper displays distinctive characteristics with high reflectance values, featuring two notable troughs at 550 nm and 1550 nm in the spectrum. Much like transparent PP, AB400L unveils distinct features solely in the SWIR range, displaying two peaks, with one exhibiting a broader profile. Additionally, it shows a nearly constant spectrum in the NIR and VIS ranges. In contrast, aluminium exhibits a consistent spectrum in the SWIR range with high reflectance values, whereas in the NIR range, it features a notable wide reduction between 800 nm to 950 nm. PET exhibits a low reflectance level, akin to transparent PP, with closely aligned spectra. This similarity can be attributed to the selected materials being both white and more transparent than the others.

It is evident from Figure 2 that each material exhibits unique characteristics in the SWIR range as opposed to the VIS range. Therefore, despite the available data in the spectral range between 450 nm to 1710 nm, in our data analysis, we exclusively considered the spectral values within the SWIR range from 810 nm to 1710 nm.

### 2.3. LED Selection Method

The LED selection was executed by simulating the system response for all the different LEDs employing the spectral data reported in Figure 2 and the spectral emissions of the LEDs as obtained from the datasheets.

**Figure 3:**
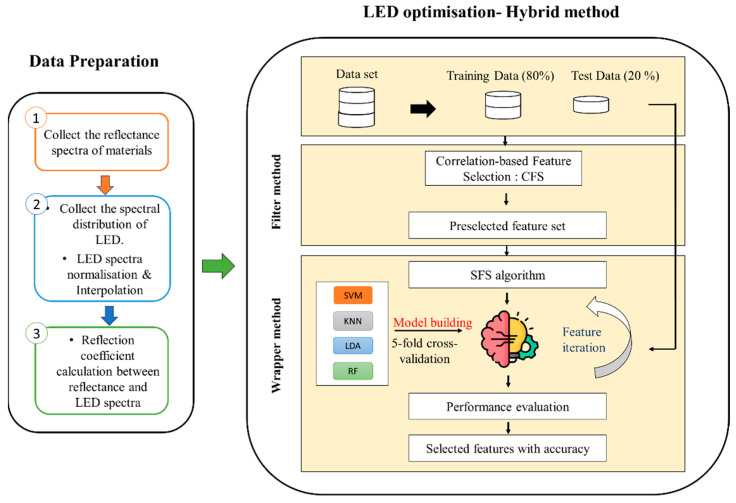
LED optimization process.

**Figure 4:**
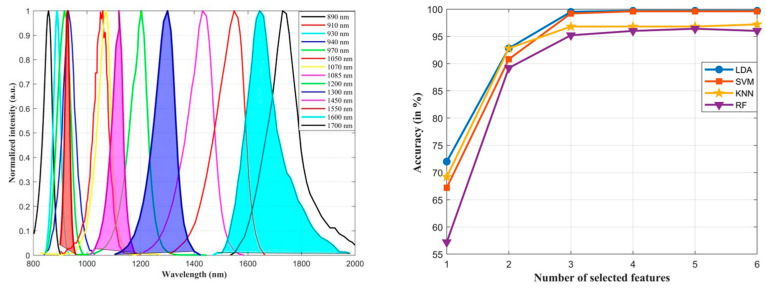
Spectral distribution of 14 LEDs with peak emission of wavelengths reported in the legend (**left**), and accuracy achieved with different classifiers for an increasing number of LEDs (**right**).

Figure 3 illustrates the process of LED selection, encompassing data preparation and optimization methods. Initially, fourteen commercially available LEDs ranging from 890 nm to 1700 nm (as shown in Figure 4) were selected, spanning the SWIR spectrum. Subsequently, a hybrid approach was employed to narrow down the selection to the top four LEDs. This hybrid method [17] was instrumental in ensuring that the eventual experimental setup would be equipped with the most fitting and efficient LEDs to achieve precise and reliable results.

The data preparation process initiated with the collection of reflectance spectra for each material using compact spectrometers, as explained in Section 2.2. Subsequently, the 14 LED spectral irradiance data were gathered from datasheets and normalized based on the intensity at the peak wavelength. The normalized irradiance spectra were then interpolated to be aligned with the wavelength range and step of the materials’ reflectance datasets. The theoretical amount of light reflected by each sample, when illuminated with a single LED, was calculated as the overlap integral of the LED irradiance spectrum and the material reflectance spectrum measured using the compact spectrometers. The coefficients derived from this process indicated the ratio of reflected light from the sample to the total incident light from the LED. This produced, for each measurement, a reduced set with 14 features that captured essential information from the reflectivity spectrum of each material. For each of the seven considered materials, the 50 collected reflectance spectra were employed for feature (i.e., LED) selection purposes.

In our hybrid approach, we adhered to a standard methodology for effective feature selection and classification model construction. We started by splitting the dataset into distinct training (80%) and testing subsets (20%). The training set became the focal point for employing the Correlation-based Feature Selection (CFS) algorithm [18], which carefully evaluates features to identify their relevance. The algorithm scores features based on their relevance, assigning priorities. Subsequently, the top 10 features with the highest scores were preselected. This subset then served as input for a Sequential Forward Selection (SFS) wrapper method [19]. Resorting to a 5-fold cross-validation, this wrapper-based optimization ensured the selection of a focused and informative feature subset comprising only 4 LEDs. Subsequently, the chosen features were utilized to construct a classification model. For a proper performance assessment, the model underwent evaluation using the untouched testing data, offering insights into its accuracy and generalization capabilities. To perform a comprehensive analysis, our methodology was applied by considering widely used classification algorithms such as Support Vector Machine (SVM) [20], K-Nearest Neighbours (KNN), Linear Discriminant Analysis (LDA), and Random Forest (RF) [21]. This standardized approach ensured a thorough exploration of feature relevance and model efficacy across diverse classifiers, contributing to the reliability and versatility of our mixed method.

After thorough evaluation, each method consistently achieved high accuracy, exceeding 95% with the set of the four selected LEDs. Notably, LDA achieved an accuracy of 99.70%, closely followed by SVM at 99.60%. We strategically selected LEDs at wavelengths of 1085 nm, 1600 nm, 1300 nm, and 910 nm due to their significant and consistent accuracy across the LDA, SVM, KNN, and RF classifiers. This choice was based on the selection of each of these LEDs within the set of the best four, for at least three classifiers out of the four considered ones, as shown in Table 1, highlighting the robustness of these LEDs in the optimization approach. Figure 4 visually presents the spectra of all 14 selected LEDs on the left side, with the filled area representing the spectra of the LEDs chosen after optimization. On the right side, the graph illustrates the LEDs alongside their corresponding accuracies at each step of the selection process for the four classification methods. Notably, as additional features were introduced, the accuracy levelled off and remained stable after selecting 6 LEDs in each method, confirming the chosen 4 LEDs were sufficient to guarantee the desired classification performance.

### 2.4. Simulation Results

**Figure 5:**
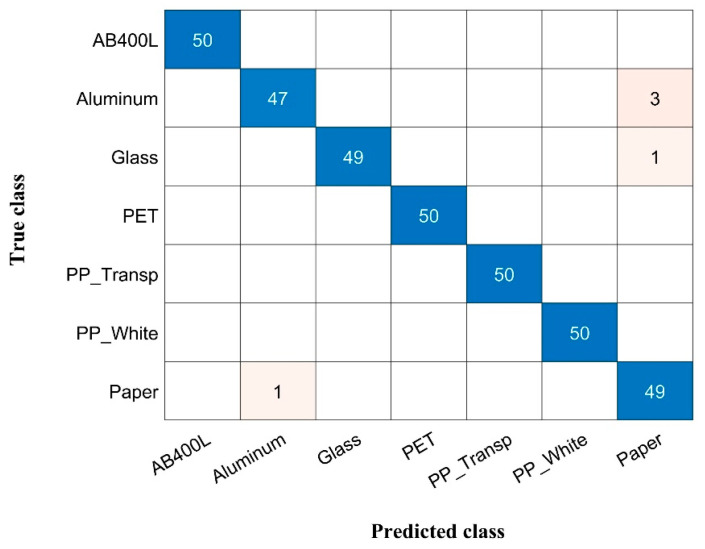
Confusion matrix obtained during the feature selection process to simulated LED data.

To provide further details on the performed analysis, and to assess the robustness of our proposed spectral acquisition and classification analysis using the selected four features (LEDs), we report in Figure 5 the confusion matrix obtained considering the LDA classifier, which demonstrated superior performance compared to the SVM, KNN, and RF algorithms. Notably, the overall accuracy rate reached 98.6%, with only a minor misclassification observed for aluminium, glass, and paper.

### 2.5. Methodology

**Figure 6:**
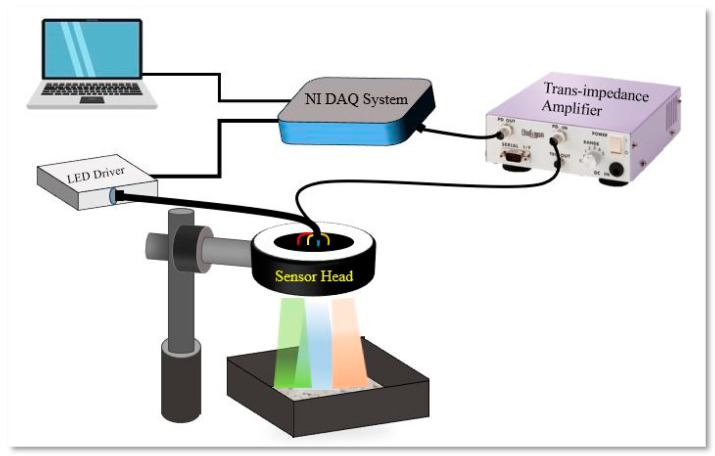
Schematic representation of the system set-up.

Following the analysis performed to select the best four LEDs, an experimental setup was built, as depicted in the schematic block diagram shown in Figure 6. This system was composed of three main components: a sensor head, an analogue front-end, and a multifunction interface connected with a computer. The sensor head was housed in a 3D-printed case with dimensions of 5 cm in diameter and 1.5 cm in height. Within this casing, there was a germanium photodetector surrounded by four strategically positioned LEDs. To prevent direct illumination, the LEDs were evenly spaced. These LED light sources are commercially available and come equipped with epoxy and glass lenses, measuring 4 mm in diameter, and have a maximum current of 25 mA. All the LEDs were biased with a custom circuit in order to provide a mean optical power of 2 mW ± 10%. The germanium photodiode operated within the spectral range of 700–1800 nm, with a peak responsivity of 0.85 A/W at 1550 nm.

The analogue front-end of the system consisted of a 4-channel current amplifier responsible for driving the LEDs and a variable-gain transimpedance amplifier designed for the photodiode. This analogue front-end was connected to a computer through a multifunction input/output interface, leveraging the National Instruments DAQ USB6009 (Austin, TX, USA). The USB6009 facilitated the sequential activation of the LED current drivers and performed analogue-to-digital conversion of the output from the photodiode amplifier. This conversion was achieved at a precision of 14 bits with a sample rate of 1 kS/s. To streamline the measurement process, a user-friendly graphic interface, developed using LabVIEW (version 2023.Q1), was employed. This interface allowed for the management of measurements, the real-time monitoring of each light source’s status, the configuration of measurement parameters (such as sampling rate, number of readings to be averaged, duration of LED illumination, and measurement timing), and the display and storage of sensor readings.

Each material sample was placed and stored in a separate black box. The samples underwent exposure to each LED light source for a duration of 10 ms. During this time, reflectance values were captured and used to calculate the ratio of reflected light to incident light. The total incident light amount was pre-calibrated and stored in a look-up table. The system output was presented graphically in LabVIEW. Maintaining a uniform illumination of the sample was ensured by placing the sensor at a fixed distance of 7 cm. A 5 V power supply was utilized to power the entire system which, along with the sample under measurement, was enclosed in a dark box. To enhance measurement accuracy, each sample was measured 10 times and averaged. Before each reading, the sample box was shaken to minimize artifacts associated with preferential reflection angles from the grains.

Upon exposure to each LED, the sample material underwent partial reflection of the incident optical radiation. The diffused reflection was captured by the photodiode, generating a photocurrent that was proportional to the optical power. This photocurrent was then amplified and converted to a voltage using the trans-impedance amplifier before being acquired by the DAQ. This acquisition process was repeated for all the different LEDs, resulting in a set of 4 values. To account for variations in the emission intensity of the 4 LEDs, all measurements were normalized with respect to a calibration dataset. This dataset was obtained by substituting the sample with a metal mirror during the calibration process. The sample’s reflectivity was calculated using Equation (1), where V0(λ) represents the voltage value obtained during the calibration with a flat mirror (proportional to the incident light intensity), and Vs(λ) is the voltage acquired while illuminating the sample under analysis (proportional to the reflected light intensity).

R(λ) = Vs(λ)/V0(λ)(1)

The measurements were conducted on the seven considered materials over a period of three days. Specifically, 50 spectra were collected from each material daily, amounting to a total of 350 spectra per day. Across the entire study, 1050 spectra were gathered, creating a comprehensive dataset that encompassed a broad range of variations and measurements for subsequent analysis. This extensive dataset enabled the consideration of the potential impact of environmental conditions on the proposed setup. In the subsequent section, various classifiers were employed on the collected data. The objective was to identify the most effective approach and to explore the relationship between achievable performance and the intra-class variability observed in the conducted measurements.

## 3. Results

The collected data were classified using various methods through a MATLAB (version 2022a) script. Table 2 below presents the classification accuracy achieved on each of the three days, employing an 80%–20% division for training and test data, and the four considered classification methods. Better results are typically obtained when using an SVM classifier, which provides the best average performance when considering data from all the three different acquisition sessions.

Overall, classification performances were similar for the different days, but some slight differences could be observed, with data from day 3 providing the worst classification results irrespective of the classification algorithm. This behaviour was expected, since we purposely did not control environmental conditions (e.g., temperature, room illumination, relative humidity) during measurements. It is therefore evident that the specific acquisition conditions may have had a non-negligible impact on the reliability of the measurements and on the resulting classification performance.

For a better assessment of the actual capabilities of the proposed system and to enhance its generalization capabilities, additional data analysis was conducted by splitting the collected samples according to two distinct modalities, that is, day-wise split and random split. The day-wise split means that data from one specific day were exclusively assigned for training, and data from another day were reserved for testing. This approach allowed for a targeted assessment of the model’s performance on unseen data from a different day, providing insights into its ability to generalize and handle variations specific to distinct timeframes. Evaluating the model under such day-wise conditions helps gauge its adaptability to changing circumstances and ensures a comprehensive understanding of its performance across different temporal contexts. The random split modality implies that the data obtained during the 3 days are mixed and then randomly divided into training (80%) and test (20%) samples in a 5-fold cross validation. Random split helps ensure a representative distribution of data across training and test sets, reducing bias and enhancing the model’s generalization to unseen data.

**Figure 7:**
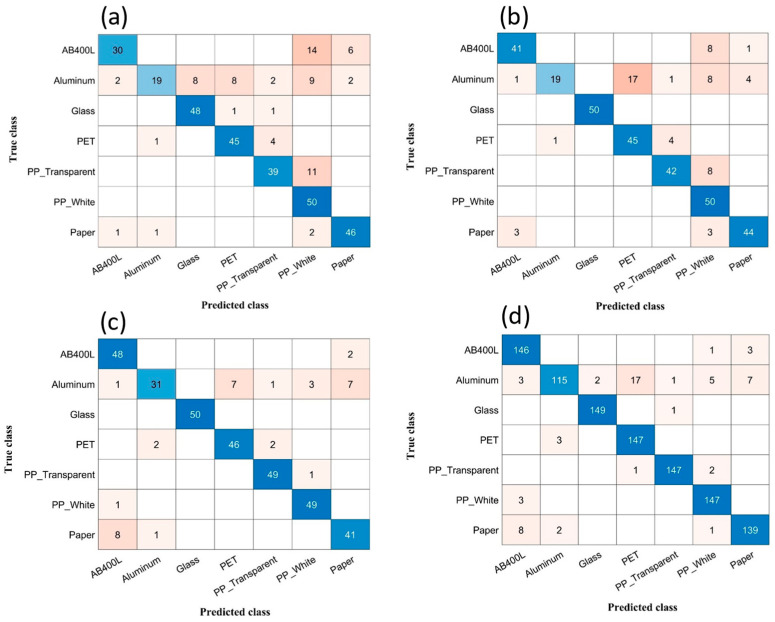
(**a**) Confusion matrix of 1st (training) vs. 2nd (test) acquisition sessions for LDA classifier. (**b**) Confusion matrix of 1st (training) vs. 3rd (test) acquisition sessions for SVM classifier. (**c**) Confusion matrix of 3rd (training) vs. 2nd (test) acquisition sessions for SVM classifier. (**d**) Confusion matrix of shuffled data acquisition sessions for SVM classifier.

Figure 7a–d show the confusion matrices associated with four out of the seven considered scenarios, that is, 1st vs. 2nd day (with LDA classifier), 1st vs. 3rd day (with SVM), 3rd vs. 2nd day (SVM), and shuffled data (SVM). The accuracies achieved in all considered tests are detailed in Table 3, where the results related to the conditions considered in Figure 7 are marked in bold. When checking the reported confusion matrices, certain noteworthy misclassifications are evident. Figure 7a reveals a significant error rate, i.e., 62%, in classifying aluminium with other materials. AB400L follows with a 40% misclassification, and PP (transparent) exhibits a 22% error rate. In Figure 7b, a persistent 62% misclassification is observed between aluminium and other materials, accompanied by a 16% misclassification between PP (transparent) and AB400L, emphasizing the complexity of accurate classification for these material pairs. Figure 7c,d continue to show a consistent trend of high misclassification for aluminium materials with other substances. Notably, in Figure 7c, there is a 38.0% misclassification with other materials, and in the shuffled dataset, a similar pattern is observed with a 23% misclassification rate. These findings highlight the difficulty in accurately categorizing aluminium using classification models applied to training/test data collected in different scenarios, which significantly impacts the overall accuracy. On the other hand, consistently high accuracy, close to 100%, is achieved for both glass and PP (white) across all the considered situations. These results suggest that these two materials have highly characterizing spectral signatures that allow effective differentiation within a classification framework.

**Figure 8:**
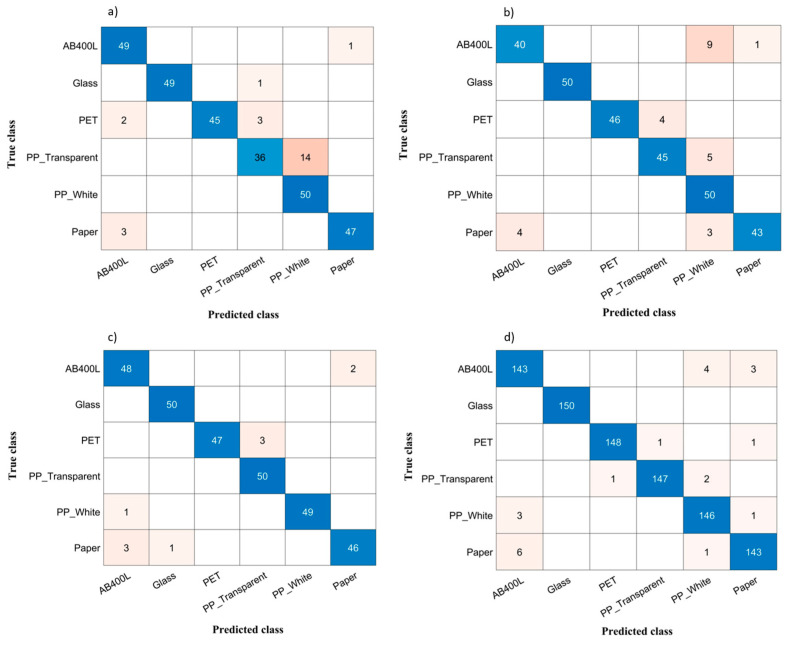
(**a**) Confusion matrix of 1st (training) vs. 2nd (test) acquisition sessions, without aluminium, for LDA. (**b**) Confusion matrix of 1st (training) vs. 3rd (test) acquisition sessions, without aluminium, for SVM. (**c**) Confusion matrix of 3rd (training) vs. 2nd (test) acquisition sessions, without aluminium, for SVM. (**d**) Confusion matrix of shuffled data acquisition sessions, without aluminium, for SVM.

The misclassification of aluminium in comparison to other materials can be attributed to the distinct surface characteristics of the materials, where one side exhibits a shiny appearance while the other is coloured. Notably, these aluminium samples were sourced from used soft drink cans obtained from the environment. To address this challenge, we opted to exclude aluminium from our dataset and focused on classifying the remaining materials. The results revealed a promising improvement in our data analysis. Actually, Table 3 outlines a comparison of the performance obtained when employing SVM and LDA classifiers, and both with and without aluminium, for all the considered data divisions. The findings from Table 3 clearly indicate that the dataset excluding aluminium exhibits substantial improvements across all scenarios. Particularly noteworthy is the fact that, out of the seven considered scenarios, five of them consistently provided overall classification accuracy above 90% without aluminium. Figure 8a–d show the confusion matrices obtained when excluding aluminium for the four scenarios considered in Figure 7. Actually, the considered scenarios, with the associated classifiers, are those showing the largest differences between considering or excluding aluminium.

The observed improvement underscores the positive impact of excluding aluminium on the model’s ability to accurately classify materials in various scenarios. Likewise, data shuffling facilitates improved the training of the classifier, enhancing its generalization capability and resulting in an overall higher accuracy of 94.3% when considering all materials, with a further improvement to 97.0% when excluding aluminium, further highlighting the positive impact of data shuffling on the model’s performance. Finally, it can be seen that the performance of the SVM classifier is consistently higher than that attained by LDA.

Despite removing aluminium from the dataset, the confusion matrices in Figure 8 highlight significant misclassifications, particularly among AB400L, PP (transparent), and paper. The classification models encounter challenges in distinguishing these three materials, particularly in the case of PP (transparent), which consistently experiences minor misclassifications with PP (white) due to their shared material properties. Glass, on the other hand, proves to be the easiest material to classify, achieving almost 100% accuracy across all cases, thanks to its distinctively low reflectance values. Furthermore, PP (white) stands out as another material with high accuracy within this system setup, attributed to its distinctive high reflectance characteristics, as evident in Figure 1, which depicts the continuous spectra of the materials. While our current system setup with 4 LEDs has demonstrated good performance in material classification, our previous analysis using a larger number of LEDs revealed superior classification for “critical” materials. This underscores the significance of achieving finer spectral discrimination for enhanced results in material classification. The utilization of a more extensive set of LEDs seems to boost the model’s capacity to differentiate and classify materials, highlighting the importance of spectral precision in achieving accurate results, particularly for critical materials.

## 4. Conclusions

This paper introduces a cost-effective approach for waste material classification through discrete optical analysis in the short-wave infrared (SWIR) range. The system integrates four LEDs and a single photodetector, controlled by basic electronic drivers and a transimpedance amplifier, respectively. The conditioned photodetector signal is acquired via a commercial data acquisition interface. With respect to our previous work using 10 LEDs and a 98% accuracy rate, our new feature selection method focuses on four LEDs, attaining 97% accuracy with support vector machines. The system is applied to classify diverse materials, including glass, paper, and three types of plastic. Challenges arise in classifying aluminium due to its reflective properties and the presence of plastic coatings on the samples.

The proposed system should be interpreted as a proof-of-concept and has been tested with a small number of samples and in a reduced complexity framework. More specifically, no coloured plastics were employed, and all the samples shared similar surface characteristics in terms of roughness. In order to assess the real extent of the proposed classification approach, further systematic analyses including samples with different colours and surface characteristics should be executed. However, it should be considered that, apart from black pigments, plastic colourings do not typically show any peculiar absorption or reflection in the SWIR range. In addition, the scattering characteristics of a certain material depend both on its surface roughness and on its complex refractive index; thus, different finishes can act as confounding parameters in the classification system, but this issue could be addressed by increasing the amount of samples and data employed for the training.

It may also be noted that, due to the employment of a single photodetector, our system may fail in the classification of samples made of mixed materials (e.g., glass bottles with plastic or metallic caps) or when dealing with very small samples with respect to the field of view of the detector. A straightforward approach to improve the performance of the system consists of the implementation of an imaging sensor as a replacement for the single photodetector, thus allowing for the acquisition of images with embedded spectral data. However, this performance enhancement would come at the cost of a more expensive measurement set-up. Despite such constraints, our research demonstrates that the proposed system is promising and highlights the potentialities of discrete spectrometric analysis for material recognition.

In summary, our research contributes to the development of an affordable and reliable system for material classification. While refining the accuracy of aluminium classification remains a challenge, our system demonstrates significant potential for accurately categorizing plastic, paper, and glass waste materials.

## Figures and Tables

**Table 1:** Ranked list of LEDs (expressed in terms of emission peak wavelength, in nm) selected for each classifier.

Methods	LED 1	LED 2	LED 3	LED 4
SVM	1085 nm	1600 nm	910 nm	1300 nm
KNN	1085 nm	910 nm	1600 nm	1300 nm
LDA	1085 nm	1600 nm	1450 nm	930 nm
RF	1085 nm	910 nm	1450 nm	1300 nm

**Table 2:** Classification accuracies achieved using four optimized LEDs across four different classification methods over the considered three-day dataset. Values in bold indicate the best results obtained for each acquisition session.

Data Set	SVM	LDA	KNN	RF
1st day	95.7%	**96.0%**	94.3%	92.9%
2nd day	**94.6%**	93.4%	92.6%	88.6%
3rd day	**93.4%**	92.9%	89.7%	90.0%

**Table 3:** Comparative analysis of classification accuracy using SVM and LDA methods in different data sets. Values in bold indicate the scenarios considered in Figure 7 and Figure 8.

Scenario	All Materials (SVM)	Without Aluminium (SVM)	All Materials (LDA)	Without Aluminium (LDA)
1st (Training) vs. 2nd (Testing)	79.4%	87.3%	**79.0%**	**92.0%**
1st (Training) vs. 3rd (Testing)	**83.0%**	**91.3%**	67.7%	88.7%
2nd (Training) vs. 1st (Testing)	76.3%	82.7%	66.6%	83.0%
2nd (Training) vs. 3rd (Testing)	80.0%	91.7%	81.4%	88.0%
3rd (Training) vs. 1st (Testing)	86.0%	95.3%	73.0%	87.7%
3rd (Training) vs. 2nd (Testing)	**82.6%**	**90.0%**	74.0%	89.7%
Shuffled data	**94.3%**	**97.0%**	93.0%	96.0%

## Data Availability

The data presented in this study are available on request from the corresponding author. The data are not publicly available due to their large dimensions.
